# NSGA-II-Based Multi-Objective Optimization of Fused Filament Fabrication Process Parameters for TPU Parts with Chemical Smoothing

**DOI:** 10.3390/polym18030391

**Published:** 2026-02-01

**Authors:** Lokeshwaran Srinivasan, Lalitha Radhakrishnan, Ezhilmaran Veeranan, Faseeulla Khan Mohammad, Syed Quadir Moinuddin, Hussain Altammar

**Affiliations:** 1 Department of Manufacturing Engineering, College of Engineering Guindy, Anna University, Chennai 600025, India; 2 Department of Mechanical Engineering, College of Engineering, King Faisal University, Al-Ahsa 31982, Saudi Arabia

**Keywords:** additive manufacturing, fused filament fabrication, thermoplastic polyurethane, surface roughness, chemical smoothing, tetrahydrofuran

## Abstract

In this study, thermoplastic polyurethane (TPU) parts were fabricated using fused filament fabrication (FFF) by varying key process parameters, namely extruder temperature (210–230 °C), layer thickness (200–400 µm), and printing speed (30–50 mm/s). A Box–Behnken experimental design was used to systematically evaluate the combined influence of these parameters on surface roughness (R_a_), dimensional deviation (DD), and ultimate tensile strength (UTS). After fabrication, all specimens were subjected to a Tetrahydrofuran (THF)-based chemical smoothing process to modify surface characteristics. Surface roughness measurements showed a substantial reduction after chemical smoothing, with values decreasing from an initial range of 13.17 ± 0.21–15.87 ± 0.23 µm to 4.01 ± 0.18–7.35 ± 0.16 µm, corresponding to an average decrease of approximately 50–72%. Dimensional deviation improved moderately, from 260–420 µm in the as-printed condition to 160–310 µm after post-processing, representing a reduction of about 20–38%. Mechanical testing revealed a consistent increase in UTS following chemical smoothing, with values improving from 30.24–40.30 ± 0.52 MPa to 33.97–47.94 ± 0.36 MPa, yielding an average increase of approximately 10–24%. Then, the experimental data were used for multi-objective optimization of the FFF process parameters, using a non-dominated sorting genetic algorithm (NSGA-II) implemented in Python 3.11, to identify best parameter combinations that provide a balanced surface quality, dimensional accuracy, and mechanical performance.

## 1. Introduction

According to ASTM ISO 52900:2022 [1], Material Extrusion (MEX), commonly referred as fused filament fabrication (FFF), is an additive manufacturing process in which thermoplastic material is selectively extruded through a nozzle to fabricate parts directly from digital models without the need for tooling [2,3]. Initially developed for rapid prototyping, FFF has used as a practical manufacturing method for producing functional components across industries such as automotive, healthcare, consumer products, and industrial tooling [4]. The process involves the extrusion of thermoplastic filaments through a heated nozzle, followed by layer-by-layer deposition based on computer-aided design (CAD) data [5].

Despite these advantages, FFF-printed components often exhibit limitations related to surface finish, mechanical performance, and dimensional consistency [6]. These issues primarily arise from the discrete nature of layer stacking, leading to a staircase effect on inclined surfaces and imperfect interlayer bonding. Surface roughness remains a significant concern, particularly for parts intended for direct use, as it affects not only aesthetic quality, but also functional aspects such as friction, wear, fatigue behaviour, and compatibility with coatings or mating components. In addition, the mechanical properties of FFF parts are strongly influenced by interlayer adhesion, which is governed by thermal and material deposition conditions during printing [7].

Extensive research has been conducted to understand the influence of FFF process parameters on part quality, particularly for commonly used rigid polymers such as polylactic acid (PLA) and acrylonitrile butadiene styrene (ABS) [8]. Parameters including extruder temperature, layer thickness, printing speed, infill density, raster angle, and build orientation have been shown to significantly affect surface roughness, tensile strength, and dimensional accuracy [9]. However, findings from rigid materials cannot be directly transferred to flexible polymers, as their flow behavior, bonding mechanisms, and deformation characteristics differ substantially.

Thermoplastic polyurethane (TPU) has gained increasing attention in FFF due to its flexibility, abrasion resistance, and durability [6]. These properties make TPU suitable for applications such as wearable devices, soft tooling, flexible fixtures, seals, and damping components [10,11,12]. Nevertheless, printing TPU using FFF presents additional challenges, including sensitivity to processing conditions and difficulty in achieving a balance between surface quality and mechanical integrity [13]. Surface irregularities are often more pronounced in flexible materials, and inadequate interlayer bonding can adversely affect tensile performance [14,15].

In addition to process parameter optimization, post-processing techniques are commonly employed to improve the quality of FFF-printed parts [16]. Among these, chemical smoothing has emerged as an effective method for reducing surface roughness by softening and reflowing the outer polymer layers. Solvent-based treatments have been widely studied for rigid thermoplastics; however, their application to TPU remains limited [17,18,19,20]. Furthermore, existing studies often focus either on printing parameter effects or on post-processing methods, without systematically examining their combined influence on part performance [20,21,22].

Another significant limitation in current research is the reliance on single-response optimization approaches. In practical applications, FFF components must satisfy multiple, often competing requirements, such as low surface roughness, high mechanical strength, and acceptable dimensional accuracy. Improving one property may degrade another, making single-objective optimization insufficient for real-world applications [23,24]. Therefore, a multi-objective optimization framework is required to identify balanced process parameter combinations.

Non-dominated sorting genetic algorithm (NSGA-II)-based optimization techniques are well suited for such problems, as they allow simultaneous optimization of multiple responses without the need to assign subjective weighting factors [25,26]. NSGA-II identifies Pareto-optimal solutions, providing a set of trade-off parameter combinations from which designers and manufacturers can select based on specific application requirements. Despite its relevance, NSGA-II-based optimization has not been widely applied to FFF-printed TPU components, particularly in studies that account for both printing parameters and post-processing effects.

In this context, the present study systematically investigates the influence of key FFF process parameters on the surface roughness (R_a_), dimensional accuracy (DD), and ultimate tensile strength (UTS) of TPU parts, followed by chemical smoothing as a post-processing step. The generated experimental data are intended to support multi-objective optimization using NSGA-II, enabling the identification of optimized parameter sets that balance surface quality and mechanical performance. The outcomes of this work are expected to contribute to the development of practical processing guidelines for manufacturing functional TPU components using FFF.

## 2. Materials and Methods

### 2.1. Material Extrusion Process Parameter Selection

The filament used in this study is eSUN TPU-95A, a polyester-based thermoplastic polyurethane supplied with a nominal filament diameter of 1.75 mm, with a Shore hardness of 95A. TPU was selected due to its flexibility, abrasion resistance, and relevance for functional applications requiring elastic behavior. The FFF process parameters were selected based on insights from existing literature and preliminary pilot experiments conducted by the authors, and considering their established influence on material deposition, interlayer bonding, and surface formation [27]. Three key process parameters were selected for investigation: extruder temperature, layer thickness, and printing speed. Extruder temperature controls the melt flow behavior and interlayer diffusion, while layer thickness governs surface resolution and layer stacking effects. Printing speed influences material deposition consistency and bonding time between successive layers. Each parameter was varied at three levels within the recommended processing range of TPU to ensure stable printing conditions.

### 2.2. Design of Experiments

A Design of Experiments (DOE) approach was adopted to systematically study the combined effects of the selected parameters. A Box–Behnken design under the response surface methodology (RSM) framework was employed due to its efficiency in evaluating interaction and quadratic effects with a reduced number of experimental runs. The design consisted of 15 experimental combinations with three input factors at three levels [28]. The independent variables were extruder temperature (210–230 °C), layer thickness (200–400 µm), and printing speed (30–50 mm/s). The output responses considered in this study were surface roughness (R_a_), average dimensional deviation (Avg. DD), and UTS. The DOE approach enabled the generation of structured data suitable for subsequent statistical analysis and multi-objective optimization.

### 2.3. Fabrication of Specimens

**Figure 1:**
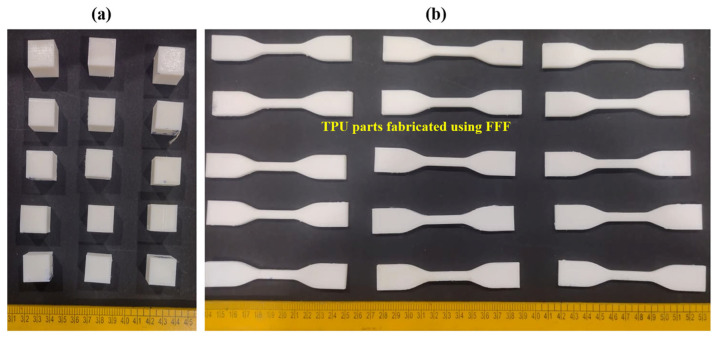
Fabricated TPU test specimens for (**a**) cube samples for surface roughness and dimensional deviation measurements and (**b**) tensile specimens for measuring ultimate tensile strength (UTS).

All specimens were fabricated using a material extrusion-based desktop 3D printer (Flashforge Finder, FlashForge Corporation, Jinhua, Zhejiang, China) equipped with a single extruder and a nozzle diameter of 600 µm, as shown in Figure 1. To ensure stable material deposition and consistent interlayer bonding, the maximum layer height was selected below the nozzle diameter, following established material extrusion guidelines. Accordingly, the layer thickness in this study was varied between 200 µm and 400 µm, remaining within the practical limits of the nozzle geometry. The printer used in this work supports an extrusion temperature of up to 240 °C, which is compatible with the processing requirements of TPU-95A filament. The motion system provides controlled positioning along the X, Y, and Z axes, enabling consistent layer generation during printing. The printing speed was varied within the range of 30–50 mm/s, which falls within the operational capability of the printer and allows evaluation of its influence on part quality.

Test specimens were modelled using CAD software, (SOLIDWORKS 2019) and exported in STL format. All specimens were sliced using FlashPrint 3.10 software. A 100% infill density with a line infill pattern was used to obtain a fully solid internal structure. The perimeter count was set to two, and the infill–perimeter overlap was fixed at 30% to ensure adequate bonding between the outer walls and the internal structure. These slicing parameters were kept constant for all samples to maintain uniform deposition conditions and enable consistent evaluation of the selected process parameters. The top and bottom regions of all specimens were generated using three solid layers each, with layer thickness corresponding to the selected printing condition (200–400 µm). All specimens were fabricated in a fixed flat orientation, with the longitudinal axis aligned parallel to the build plate (XY plane), and this build orientation was maintained consistently for tensile, surface roughness, and dimensional accuracy specimens across all experimental runs. This configuration provided continuous outer surfaces and limited internal exposure during chemical smoothing, thereby influencing surface modification and dimensional stability. The top and bottom layer settings were kept constant for all samples to ensure consistency across experiments.

Tensile test specimens were designed according to ASTM D638 standards [29] (Type IV), while cube samples of dimension 10 × 10 × 10 mm^3^ were fabricated for surface roughness and dimensional measurements. Printing was carried out according to the parameter combinations specified by the DOE. No additional surface finishing or mechanical treatment was applied before post-processing to preserve the as-printed characteristics for comparison.

### 2.4. Chemical Smoothing

**Figure 2:**
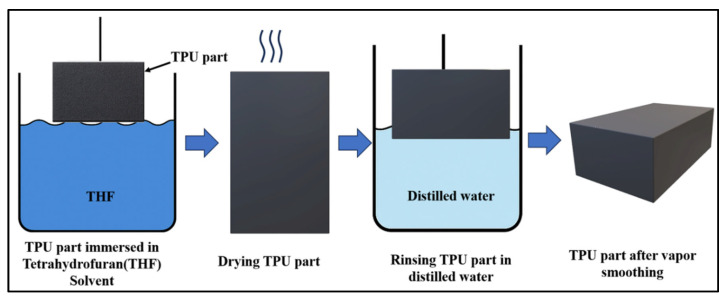
Schematic representation of the immersion-based chemical smoothing process using THF.

A Chemical Smoothing (CS) post-processing technique was carried out using an immersion-based solvent treatment with THF to improve the surface quality of the FFF-printed TPU parts, as shown in Figure 2. THF is a polar organic solvent capable of interacting with polyurethane chains by partially dissolving the surface layer without significantly affecting the bulk material. This controlled surface interaction enables polymer +chain mobility at the outer layers, resulting in surface reflow and smoothing.

The chemical smoothing process was performed using tetrahydrofuran (THF) as the solvent under controlled conditions. The specimens were fully immersed in pure THF (analytical grade of 99.5% pure) at ambient temperature (25 ± 2 °C) under static conditions, without agitation, to ensure uniform solvent interaction with the printed surfaces.

The immersion time was fixed at 3 min following preliminary process evaluation, where shorter exposure durations produced limited surface modification, while longer immersion times led to noticeable changes in part geometry. The selected duration provided sufficient surface modification while maintaining dimensional stability.

During CS, THF penetrates the outermost layers of the TPU, causing localized swelling and partial dissolution of surface asperities formed during layer-by-layer deposition. CS redistributes polymer material across the surface, reducing the visibility of layer lines and surface irregularities. After the CS step, the specimens were carefully removed from the solvent and allowed to dry at ambient laboratory conditions for 2 h. The drying stage enables gradual solvent evaporation, allowing the softened surface layers to solidify and stabilize without inducing internal stresses. Controlled drying is essential to prevent surface defects such as blistering or uneven re-solidification. Following drying, the samples were rinsed with distilled water to remove any residual solvent or surface contaminants. This cleaning step ensures that no solvent residues remain on the part surface, which could otherwise influence subsequent mechanical testing or surface measurements. The specimens were then allowed to air-dry completely before characterization.

**Figure 3:**
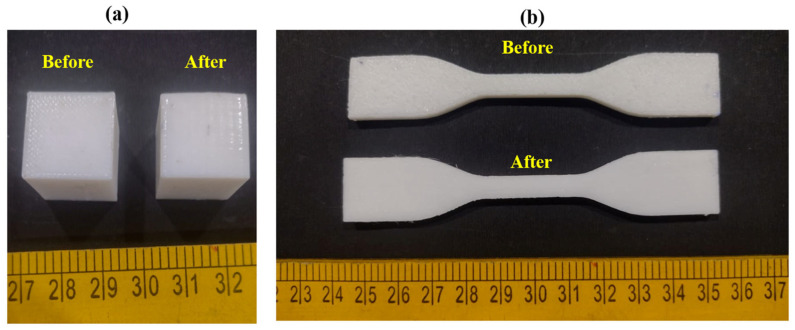
TPU specimens before and after post processing with chemical smoothing process: (**a**) cube samples and (**b**) tensile specimens.

The immersion-based THF treatment primarily modifies the surface and near-surface regions of the TPU parts, while the internal structure remains unchanged. As a result, this post-processing method improves surface finish and interlayer bonding near the surface without causing significant dimensional distortion or degradation of the bulk material, as observed in Figure 3. The same post-processing conditions were applied to all specimens to maintain consistency and to isolate the influence of the FFF process parameters on the final part performance.

### 2.5. Surface Measurement and Mechanical Testing

Surface roughness measurements were carried out using a contact-type surface roughness tester (Mitutoyo Surftest SJ-210 contact profilometer, Mitutoyo Corporation, Kawasaki, Japan) equipped with a diamond stylus having a tip radius of 2 µm. The measurements were performed with a traverse speed of 0.5 mm/s. A cutoff length of 0.8 mm was selected, and the surface roughness parameter R_a_ was evaluated over an evaluation length of 4.0 mm, corresponding to five consecutive cutoff lengths. A Gaussian filter was applied to separate roughness from waviness components. For each specimen, ten measurements were taken at different locations on the same surface, and the average value was used for analysis to minimize local variability. The average surface roughness (R_a_) was recorded by tracing the stylus along the printed surface in a direction perpendicular to the layer lines.

Mechanical testing was carried out using a universal testing machine to determine the UTS of the specimens. Tensile tests were conducted according to ASTM D638 at a constant crosshead speed. Tests were performed on specimens both before and after CS to assess the influence of post-processing. The tests were conducted at a constant crosshead speed of 1 mm/min and the gauge length was maintained at 50 mm, which is suitable for flexible polymer materials such as TPU. Ultimate tensile strength values were obtained from the stress–strain curves generated during testing.

Dimensional accuracy was evaluated using a digital caliper with a resolution of 10 µm. Measurements were taken along the X, Y, and Z directions five times, and the average value was used to calculate dimensional deviation. The DD was calculated as the difference between measured and nominal values.

### 2.6. NSGA-II-Based Multi-Objective Optimization

To identify optimal processing conditions that consider multiple performance criteria, NSGA-II was employed [30]. The experimental data obtained from the DOE served as the input dataset for optimization. The objectives considered were the minimization of surface roughness and dimensional deviation, along with the maximization of UTS [31]. NSGA-II was used to generate a set of Pareto-optimal solutions representing trade-offs among the selected responses. This approach avoids the need for predefined weighting factors and allows selection of parameter combinations based on specific application requirements. The optimized parameter set guides the balancing of surface quality and mechanical performance in FFF-printed TPU components.

## 3. Results and Discussion

### 3.1. Statistical Analysis Using Response Surface Methodology

**Figure 4:**
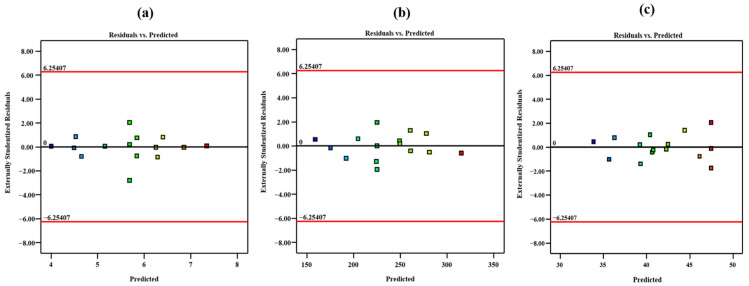
Plot of residuals versus predicted values for (**a**) surface roughness, (**b**) Avg. DD and (**c**) UTS.

The experimental results obtained for surface roughness (R_a_), average DD, and UTS of the FFF-fabricated TPU specimens were analyzed using RSM in Design Expert 13 software. The measured responses corresponding to each combination of process parameters were used to establish statistical models describing the relationship between input factors and output responses (Table 1). All statistical analyses were performed using a 95% confidence level (*p* ≤ 0.05). Analysis of variance (ANOVA) was applied to evaluate the significance of individual terms, the adequacy of the developed regression models, and the presence of lack-of-fit (Table 2). The statistical evaluation confirmed that the selected quadratic models adequately represent the experimental data within the parameter range studied. Model adequacy was verified through statistical indicators such as the coefficient of determination (R^2^) and signal-to-noise ratio (Adequate Precision > 4), which indicated good agreement between predicted and measured values (Table 3). The adequacy of the developed regression models was verified using diagnostic plots. Normal probability plots of the residuals were examined to assess the assumption of normality, while plots of residuals versus predicted values were used to evaluate homoscedasticity (Figure 4). The residuals were observed to be randomly distributed around zero with no systematic patterns, indicating that the assumptions required for regression-based analysis of variance were satisfied. Terms with negligible influence on the responses, identified through probability values exceeding the accepted threshold, were excluded to improve model clarity and predictive capability. The refined models demonstrated reliable predictive performance for surface roughness, DD, and UTS. The resulting regression equations describe the influence of process parameters and their interactions on the responses and provide a basis for subsequent optimization. The regression equations presented in this study are expressed in standardized (coded) form, where A, B, and C represent dimensionless coded variables corresponding to extruder temperature, layer thickness, and printing speed, respectively. The use of coded variables allows for direct comparison of the relative influence of each process parameter on the responses (Table 4).

### 3.2. Influence of Process Parameters on Surface Roughness, Dimensional Deviation and Tensile Strength

#### 3.2.1. Effect of Extruder Temperature

Extruder temperature showed a nonlinear influence on both dimensional deviation and UTS. At lower temperatures, insufficient material flow resulted in weaker interlayer bonding, which adversely affected mechanical performance. As the temperature increased, the improved filament fusion enhanced tensile strength due to better polymer chain diffusion across layers [27]. However, beyond an optimal temperature range, DD tended to increase slightly, likely due to excessive material flow affecting geometric stability. This behavior indicates the need for controlled temperature selection to balance mechanical performance and dimensional accuracy.

#### 3.2.2. Effect of Layer Thickness

Layer thickness was the most influential parameter affecting DD. Increasing layer thickness reduced the number of deposited layers, which amplified staircase effects and dimensional inconsistency, particularly along the build direction [32]. Lower layer thickness produced a more uniform geometry due to finer layer resolution. The influence of layer thickness on UTS was less pronounced compared to its effect on dimensional accuracy. However, thinner layers generally contributed to improved mechanical performance by increasing interlayer contact area and reducing void formation between successive layers.

#### 3.2.3. Effect of Printing Speed

Printing speed affected both DD and UTS through its control over deposition time and bonding conditions. At higher speeds, reduced contact time between deposited filaments limited interlayer diffusion, leading to lower UTS and increased variability in dimensions [27]. Lower printing speeds allowed for more stable deposition and improved bonding, resulting in enhanced mechanical performance and reduced DD. The interaction between printing speed and extruder temperature further influenced response behavior, emphasizing the need for coordinated parameter selection.

### 3.3. Effect of Chemical Smoothing on Surface Roughness, Dimensional Deviation and Tensile Strength

#### 3.3.1. Effect of Chemical Smoothing on Surface Roughness

**Figure 5:**
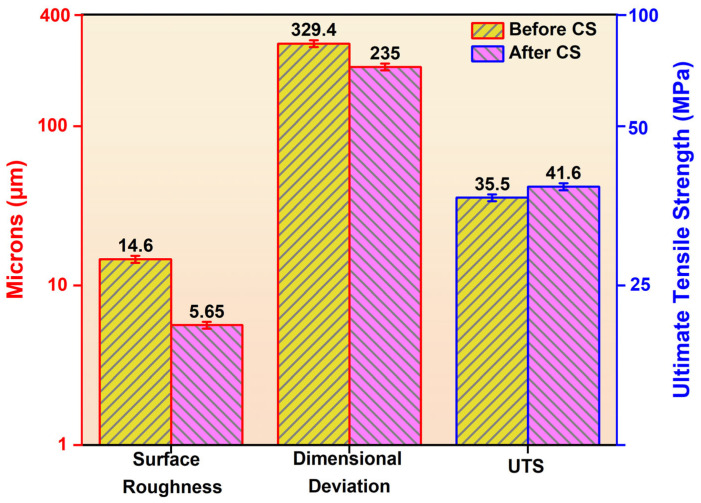
Comparison of TPU properties before and after chemical smoothing.

CS resulted in a substantial reduction in surface roughness for all experimental runs. Before post-processing, the surface roughness values ranged from 13.17 µm to 15.87 µm, reflecting the pronounced layer-line texture inherent to FFF-fabricated TPU parts. After CS, the roughness values decreased to a range of 4.01 µm to 7.35 µm, indicating effective modification of the outer surface, as shown in Figure 5.

**Figure 6:**
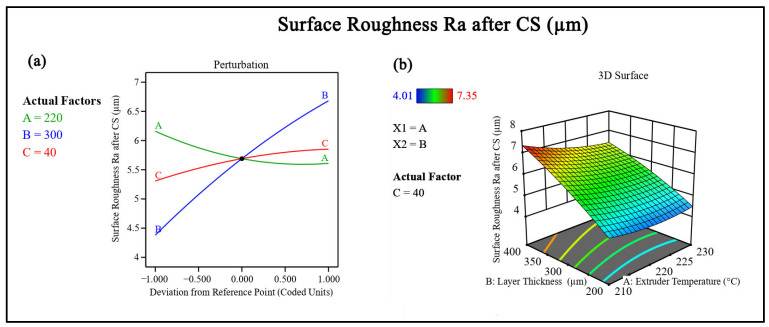
(**a**) Perturbation plot showing the influence of process parameters on surface roughness. (**b**) Response surface plot illustrating the combined effect of parameters on surface roughness.

The percentage reduction in surface roughness varied between approximately 50% and 72%, depending on the printing parameters. In Run 4, surface roughness decreased from 14.09 µm to 4.49 µm, corresponding to a reduction of about 68.1%. Similarly, Run 13 showed a decrease from 13.61 µm to 4.01 µm, representing a reduction of approximately 70.5%. Even in cases with higher layer thickness (Figure 6) and printing speed, such as Run 12, surface roughness was reduced by around 52.1%.

**Figure 7:**
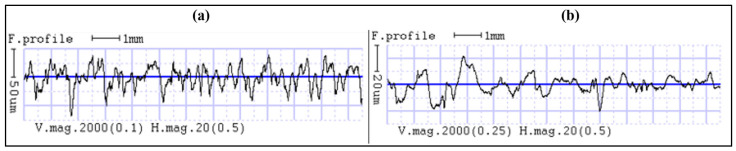
2D surface roughness profile: (**a**) Before Chemical Smoothing; (**b**) After Chemical Smoothing.

This consistent reduction can be attributed to the ability of CS to modify surface asperities formed during layer-by-layer deposition, as shown in Figure 7, where the blue mean line serves as a reference baseline and the peak–valley amplitudes relative to this line are noticeably reduced after post-processing. The treatment primarily affects the outermost layers, allowing for the redistribution of material, reducing peak-to-valley height variations without altering the internal structure.

#### 3.3.2. Effect of Chemical Smoothing on Dimensional Deviation

DD showed a moderate but consistent improvement following CS. Prior to post-processing, the average DD ranged from 260 to 420 µm, while after treatment, it was reduced to 160 to 306 µm, as observed in Figure 5.

**Figure 8:**
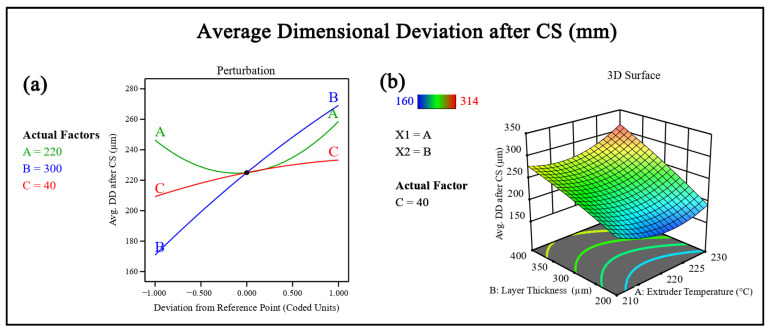
(**a**) Perturbation plot showing the influence of process parameters on dimensional deviation. (**b**) Response surface plot illustrating the combined effect of parameters on dimensional deviation.

The percentage reduction in DD ranged from 20% to 38%. For instance, in Run 1, dimensional deviation decreased from 420 to 306 µm, corresponding to a reduction of approximately 27.1%. In Run 13, the deviation reduced from 260 to 160 µm, indicating an improvement of about 38.5%. Runs with higher layer thickness values exhibited relatively smaller reductions, typically around 20–25%, as seen in Runs 7 and 12. The observed improvement is mainly due to surface-level material redistribution, which smooths sharp geometric irregularities and reduces localized dimensional offsets, as shown in Figure 8. However, since CS does not alter the internal layer stacking sequence, improvements in dimensional accuracy remain limited compared to surface roughness enhancement. All measured deviations were positive, indicating that the as-printed specimens exhibited oversizing relative to the nominal dimensions along the evaluated axes. This behavior is attributed to material deposition characteristics inherent to the material extrusion process.

Following chemical smoothing, a consistent reduction in the magnitude of the positive dimensional deviation was observed across all experimental conditions. This reduction indicates improved dimensional conformity after post-processing.

#### 3.3.3. Effect of Chemical Smoothing on Ultimate Tensile Strength

**Figure 9:**
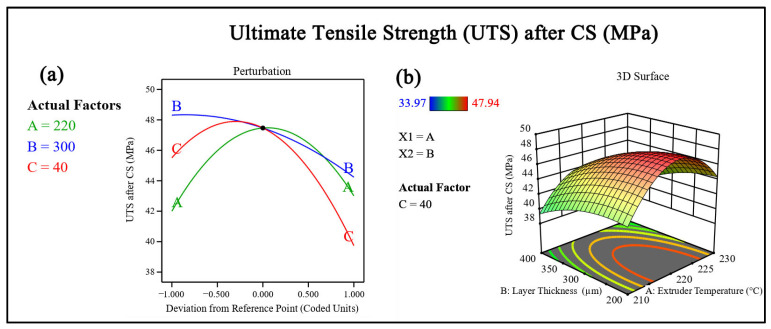
(**a**) Perturbation plot showing the influence of process parameters on ultimate tensile strength. (**b**) Response surface plot illustrates the combined effect of parameters on ultimate tensile strength.

**Figure 10:**
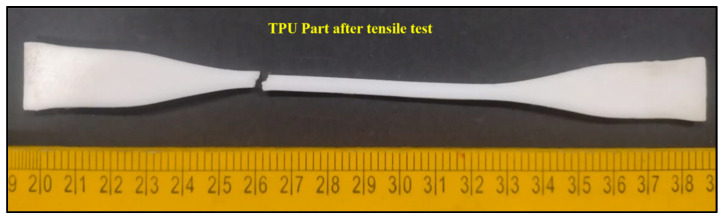
TPU tensile specimen after fracture during tensile testing of Run 10.

**Figure 11:**
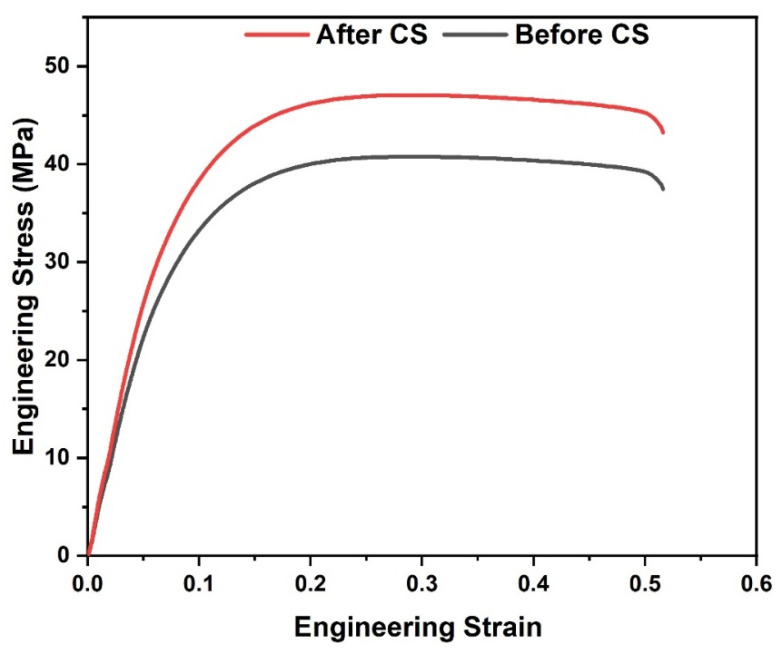
Engineering Stress–strain behavior of the TPU sample from Run 10.

UTS increased for all experimental runs after CS, indicating a beneficial influence on mechanical performance (Figure 9). Before treatment, UTS values ranged from 30.24 MPa to 40.30 MPa, whereas after smoothing, they increased to 33.97 MPa to 47.94 MPa. The percentage improvement in UTS varied between approximately 10% and 23%. For example, Run 2 exhibited an increase from 34.37 MPa to 42.54 MPa, corresponding to an improvement of about 23.8%. In Run 10 (Figure 10 and Figure 11), UTS increased from 40.30 MPa to 47.94 MPa, yielding an enhancement of approximately 18.9%. Lower improvements, around 10–12%, were observed in runs such as Run 3, where UTS increased from 30.24 to 33.97 MPa.

The improvement in UTS can be attributed to enhanced near-surface interlayer bonding and a reduction in stress concentration at surface-level defects. By smoothing interfacial irregularities, chemical treatment reduces premature crack initiation during tensile loading, leading to improved load-bearing capacity [33]. The effect of chemical smoothing on elongation at break was not considered as a primary response in the present study. However, qualitative observations during tensile testing indicated that the chemically smoothed TPU specimens did not exhibit a noticeable reduction in elongation compared to the as-printed samples. The stress–strain curves showed a similar deformation behavior before and after chemical smoothing, suggesting that the applied THF-based post-processing primarily influenced surface characteristics and interlayer bonding without significantly altering the overall ductile response of the material.

### 3.4. Multi-Objective Optimization Using NSGA-II

To identify parameter combinations that provide balanced performance, the experimental data was used for multi-objective optimization using the NSGA-II. A Python implementation of NSGA-II was employed to simultaneously minimize surface roughness and DD while maximizing UTS. The multi-objective optimization was implemented in Python using NumPy and Pandas for numerical computation and data handling, pymoo for NSGA-II–based optimization, and Matplotlib (version 3.8) for Pareto front visualization. A population size of 50 and 100 generations were selected to ensure adequate solution diversity and convergence with reasonable computational effort. Initial solutions were generated using random floating-point sampling over the defined design space. Simulated binary crossover was applied with a probability of 0.9 and a distribution index of 15 to promote effective recombination. Polynomial mutation with a distribution index of 20 was used to maintain diversity and avoid premature convergence. Selection was based on non-dominated sorting and crowding distance, ensuring a well-distributed Pareto front. Instead of selecting a single optimal solution, NSGA-II generated a set of Pareto-optimal solutions that represent trade-offs among the responses. These solutions allow selection of printing parameters based on specific application requirements, as a parameter set that prioritizes surface finish may differ from one that prioritizes mechanical strength.

The NSGA-II–based optimization results were experimentally validated (Table 5), and the best agreement between predicted and measured responses was observed for the following parameter set: 220 °C extruder temperature, 210 µm layer thickness, and 30 mm/s printing speed. The optimized process parameter combination identified using NSGA-II was also subjected to the same chemical smoothing (CS) conditions applied in this study. For this optimized condition, the untreated specimen exhibited a surface roughness of approximately 13.2 µm, an average dimensional deviation of about 260 µm, and an ultimate tensile strength of around 40.3 MPa. After chemical smoothing, the corresponding values were reduced to approximately 4.06 µm for surface roughness and 165 µm for dimensional deviation, while the ultimate tensile strength increased to about 46.34 MPa. This direct comparison between untreated and chemically smoothed specimens under optimized printing parameters clearly demonstrates the contribution of chemical smoothing to surface refinement and dimensional control, along with a moderate improvement in mechanical performance.

**Figure 12:**
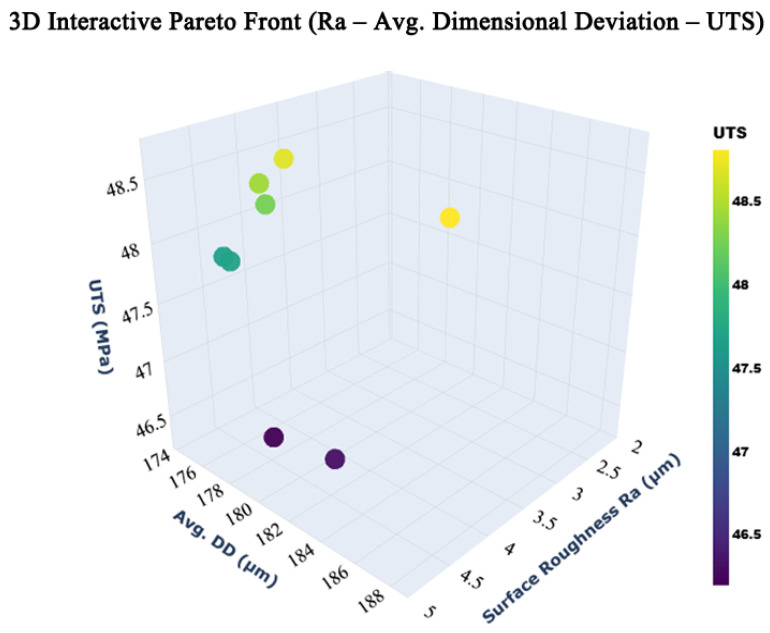
Pareto-optimal front obtained using the NSGA-II algorithm for multi-objective process parameter optimization.

The remaining optimized parameter combinations also showed similarly consistent agreement, with surface roughness typically maintained at 4.0–4.4 µm, dimensional deviation was maintained within the range of 160–180 µm, and UTS was maintained in the range of 45–47 MPa (Figure 12). Overall, the prediction error across all responses remained within approximately 2–6%, confirming the reliability of the regression models and NSGA-II framework. These findings demonstrate that TPU components fabricated using the optimized FFF parameters, and then subjected to standard chemical smoothing, can achieve stable surface, dimensional, and mechanical performance.

## 4. Conclusions

This study examined the combined influence of fused filament fabrication (FFF) process parameters and chemical post-processing on the surface quality, dimensional accuracy, and mechanical performance of TPU parts. The experimental data were further used for multi-objective optimization using a non-dominated sorting genetic algorithm. The key findings of this study are summarized as follows:

- The surface roughness of FFF-printed TPU parts was reduced by 50–72%, decreasing from 13.17–15.87 ± 0.22 µm to 4.01–7.35 ± 0.17 µm after chemical smoothing.
- Dimensional deviation showed a moderate reduction of about 20–38%, improving from 260–420 µm in the as-printed condition to 160–306 µm after post-processing.
- Ultimate tensile strength increased by a range of 10–24%, with values improving from 30.24–40.30 ± 1.52 MPa to 33.97–47.94 ± 1.36 MPa following chemical smoothing.
- The optimal FFF parameter combinations identified using NSGA-II are 220 °C extruder temperature, 210 µm layer thickness, and 30 mm/s printing speed, resulting in surface roughness, dimensional accuracy, and tensile strength prediction errors ranging from 4.14–5.69% for overall part geometry.

Therefore, the combined use of optimized FFF parameters and a fixed chemical smoothing step enables consistent improvement in the functional performance of TPU parts fabricated by FFF. Future work will focus on extending this approach to other flexible polymers, evaluating durability, and integrating broader optimization objectives to improve application-specific performance.

## Figures and Tables

**Table 1:** Experimental design, process parameter settings, and measured responses.

Run	A—Extruder Temperature (°C)	B—Layer Thickness (µm)	C—Printing Speed (mm/s)	UTS (MPa)		Surface Roughness (R_a_) (µm)		Avg. DD (µm)	
				Before CS	After CS	Before CS	After CS	Before CS	After CS
1	230	400	40	32.4	39.3	14.61	6.45	420	314
2	220	400	30	34.4	42.5	14.04	6.24	390	250
3	210	300	50	30.2	34	15.87	6.25	340	260
4	230	200	40	39.1	44.6	14.09	4.49	290	190
5	220	200	50	34.9	40.8	15.36	4.58	260	175
6	230	300	50	31.3	35.5	14.71	5.8	330	263
7	220	400	50	30.6	36.4	15.27	6.85	370	280
8	220	300	40	39.9	47	14.63	5.83	331	230
9	210	200	40	38.4	42.3	15.75	4.61	270	206
10	220	300	40	40.3	47.9	14.78	5.71	300	225
11	210	300	30	35.1	40.6	14.39	5.89	320	222
12	210	400	40	35.6	39.1	15.35	7.35	370	280
13	220	200	30	38.2	46	13.61	4.01	260	160
14	220	300	40	40.3	47.4	14.67	5.53	320	220
15	230	300	30	33	40.6	13.17	5.16	370	250

**Table 2:** ANOVA for the developed response models.

Response	Source	Sum of Squares	Degrees of Freedom	Mean Square	F-Value	*p*-Value	Remarks
UTS After CS	Model	269.65	9	29.96	227.69	<0.0001	significant
	A—Extruder Temperature	2.04	1	2.04	15.51	0.011	
	B—Layer Thickness	33.21	1	33.21	252.4	<0.0001	
	C—Printing Speed	66.59	1	66.59	506.04	<0.0001	
	AB	1.23	1	1.23	9.36	0.0281	
	AC	0.5929	1	0.5929	4.51	0.0872	
	A^2^	90.96	1	90.96	691.26	<0.0001	
	B^2^	5.26	1	5.26	39.96	0.0015	
	C^2^	86.61	1	86.61	658.24	<0.0001	
	Residual	0.6579	5	0.1316			
	Lack of Fit	0.2418	3	0.0806	0.3875	0.7771	not significant
Surface Roughness (R_a_) After CS	Model	12.23	7	1.75	157.13	<0.0001	significant
	A–Extruder Temperature	0.605	1	0.605	54.4	0.0002	
	B–Layer Thickness	10.58	1	10.58	951.32	<0.0001	
	C–Printing Speed	0.594	1	0.594	53.41	0.0002	
	AB	0.1521	1	0.1521	13.68	0.0077	
	A^2^	0.1404	1	0.1404	12.62	0.0093	
	B^2^	0.0945	1	0.0945	8.5	0.0225	
	C^2^	0.0447	1	0.0447	4.02	0.0851	
	Residual	0.0778	7	0.0111			
	Lack of Fit	0.0322	5	0.0064	0.2829	0.8895	not significant
Avg. DD After CS	Model	0.0238	9	0.0026	116.79	<0.0001	significant
	A-Extruder Temperature	0.0001	1	0.0001	4.97	0.0462	
	B-Layer Thickness	0.02	1	0.02	883.65	0.0251	
	C-Printing Speed	0.001	1	0.001	44.73	0.0049	
	AB	0.0003	1	0.0003	12.03	0.0179	
	AC	0.0005	1	0.0005	22.37	0.0052	
	A^2^	0.0016	1	0.0016	68.84	0.0004	
	C^2^	0.0002	1	0.0002	6.8	0.0478	
	Residual	0.0001	5	0			
	Lack of Fit	0.0001	3	0	0.8224	0.5896	not significant

**Table 3:** Fit Statistics.

Response After CS	R^2^	Adjusted R^2^	Predicted R^2^	Adequate Precision
UTS	0.975	0.9694	0.9565	39.9
Surface roughness (R_a_)	0.971	0.9726	0.9668	38.39
Avg. DD	0.972	0.9646	0.9595	40.17

**Table 4:** Regression equations relating process parameters to output responses after CS.

UTS	=−2473.10750 + 22.05567 A + 173.32500 B + 3.58617 C − 0.555000 AB − 0.049633 A^2^ − 119.33333 B^2^ − 0.048433 C^2^
R_a_	=+91.37385 − 0.864231 A +63.49231 B + 0.027250 C − 0.195000 AB + 0.002035 A^2^ − 15.15385 B^2^
Avg. DD	=13609.106 − 123.329 A − 2258.75 B +17.642 C +12.5 AB − 0.0625 AC + 0.279 A^2^ − 0.0337 C^2^

*Note:* A, B, and C are coded forms of Extruder Temperature, Layer Thickness, and Printing Speed, respectively.

**Table 5:** Comparison of predicted and experimental values with optimized FFF parameters.

S. No.	Extruder Temp (°C)	Layer Thickness (µm)	Printing Speed (mm/s)	Predicted Values			Experimental Values			Error Percentage (%)		
				R_a_ (µm)	Avg.DD (µm)	UTS (MPa)	R_a_ (µm)	Avg.DD (µm)	UTS (MPa)	R_a_ (µm)	Avg.DD (µm)	UTS (MPa)
1	222	220	39	4.59	187	48.7	4.44	175	45.95	3.27	6.42	5.61
2	222	210	35	4.39	178	48.7	4.2	169	46.56	4.33	5.06	4.32
3	219	220	34	4.47	178	48.2	4.24	167	45.97	5.15	6.18	4.67
**4**	**220**	**210**	**30**	**4.16**	**169**	**48.2**	**4.06**	**165**	**46.34**	**2.4**	**2.37**	**3.84**
5	220	200	30	4.24	169	47.6	4.05	162	44.91	4.48	4.14	5.69
6	219	210	33	1.29	175	47.7	4.1	167	44.91	4.46	4.16	4.49
7	223	200	37	4.28	178	48.7	4.09	170	46.56	4.33	5.06	4.65
8	218	200	36	4.27	176	48.44	4.05	166	45.97	5.15	6.18	4.61

## Data Availability

The raw data supporting the conclusions of this article will be made available by the authors on request.
